# Th17/Treg Cells Imbalance and GITRL Profile in Patients with Hashimoto’s Thyroiditis

**DOI:** 10.3390/ijms151221674

**Published:** 2014-11-25

**Authors:** Yingzhao Liu, Xinyi Tang, Jie Tian, Chenlu Zhu, Huiyong Peng, Ke Rui, Yungang Wang, Chaoming Mao, Jie Ma, Liwei Lu, Huaxi Xu, Shengjun Wang

**Affiliations:** 1 Department of Laboratory Medicine, the Affiliated People’s Hospital, Jiangsu University, Zhenjiang 212002, China; E-Mails: zjliuyingzhao@126.com (Y.L.); penghuiyong33815@163.com (H.P.); 2 Institute of Laboratory Medicine, Jiangsu Key Laboratory of Medical Science and Laboratory Medicine, Jiangsu University, Zhenjiang 212013, China; E-Mails: xinyitang0301@sina.com (X.T.); tjj850913@163.com (J.T.); zcl51234@163.com (C.Z.); j827864988@163.com (K.R.); wyg1223@126.com (Y.W.); mcm20040901@126.com (C.M.); zjflmj19780723@126.com (J.M.); 3 Department of Pathology and Centre of Infection and Immunology, The University of Hong Kong, Hong Kong 999077, China; E-Mail: liweilu@hkucc.hku.hk

**Keywords:** Hashimoto’s thyroiditis, Th17 cells, Treg cells, GITRL

## Abstract

Hashimoto’s thyroiditis (HT) is an organ-specific immune disease characterized by the presence of lymphocytic infiltration and serum autoantibodies. Previous studies have confirmed the critical role of Th17 cells in the pathopoiesis of HT patients. Additionally, regulatory T cells (Treg) display a dysregulatory function in autoimmune disease. The purpose of this study is to investigate the alteration of Th17 and Treg cells in HT patients and explore contributing factors. We found there was an increased ratio of Th17/Treg in HT patients and a positive correlation with autoantibodies (anti-TgAb). In addition, there was an increased level of GITRL, which has been demonstrated to be correlated with the increassement of Th17 cells in the serum and thyroid glands of HT patients; the upregulated serum level of GITRL has a positive correlation with the percentage of Th17 cells in HT patients. In summary, an increase in GITRL may impair the balance of Th17/Treg, and contribute to the pathopoiesis of Hashimoto’s thyroiditis.

## 1. Introduction

Human autoimmune thyroid disease (AITD) is an organ-specific immune disease characterized by autoantibodies, such as anti-thyroglobulin (Tg) antibody, anti-thyroperoxidase (TPO) antibody and anti-TSH receptor (TSHR) antibody, mainly involving Hashimoto’s thyroiditis (HT) and Graves’ disease (GD) [1,2,3].

CD4^+^T helper T cells produce large amounts of cytokines in response to antigen-specific activation. They are defined as Th1, Th2, Th17 and Tfh cells on the basis of their cytokine-expression profiles. The Th17 lineage of CD4^+^T helper cells produces interleukin 17 (IL-17) [4], a cytokine that induces the production of chemokines and antimicrobial peptides, leading to the recruitment of neutrophils and inflammation [5]. Th17 cells provide protection in certain infections but have also been linked to the development of autoimmune diseases, including autoimmune arthritis, Crohn’s disease, multiple sclerosis, dermatomyositis and so on [6,7,8,9,10]. The immune response needs to be controlled to avoid an overflowing immune response, and chronic inflammation. For that goal, there is a subset of CD4^+^T cells known to have suppressive activity and an important role in the self-tolerance, regulatory T cells (Treg cells) [11,12]. Th17 and Treg cells have opposite roles in the development of autoimmune diseases. While Th17 cells promote autoimmunity, Treg cells can control it and play a critical role in autoimmune pathogenesis by maintaining self-tolerance and controlling activation of autoreactive CD4^+^T effector cells [13]. The imbalance of Th17/Treg appears also very important in the development of these diseases [14,15].

Glucocorticoid-induced tumor necrosis factor receptor (GITR) family-related protein, also know as TNFRSF18, is a type 1 transmembrane protein with an extracellular N terminus and cytoplasmic *C* terminus [16]. GITR is expressed at different levels in resting CD4^+^ and CD8^+^T cells and is up-regulated after T-cell activation [17]. GITR is also constitutively expressed on CD4^+^CD25^+^Treg cells at high levels [18]. The natural ligand of GITR (GITRL) is predominantly expressed by antigen-presenting cells (APCs), including dendritic cells (DCs) and macrophages [19]. Engagement of GITR by GITRL abrogated the immunosuppressive function of Treg cells. Signaling cascades triggered through GITR and GITRL influence many physiologic and pathologic immune responses by regulating proliferation, differentiation, survival, and functions of immunocytes in both the innate and adaptive immune systems [20,21]. Additonally, an earlier study from our group has certificated a function of GITRL in exacerbating autoimmune arthritis via the enhancement of the expansion of Th17 cells [8].

It has been confirmed that the interaction of APCs, thyroidal follicular cells (TFCs) and autoreactive T cells results in an autoimmune response against thyroid antigens, which is mediated by Th-1 or Th-2 cells [22]. According to our previous studies, there was an increased frequency of Th17 cells in patients with Hashimoto’s thyroiditis [23,24]. Apart from the enhancement of Th17 cells, there was a reduction of Treg cells in this study. The imbalance between Th17 cells and Treg cells may influence pathology or disease outcomes in Hashimoto’s thyroiditis. We also found that the expression of GITRL was increased in HT patients, and the expression of GITRL correlated with proportions of Th17 cells.

## 2. Results

### 2.1. Enhancement of Th17 Cells in Peripheral Blood from HT Patients

**Figure 1:**
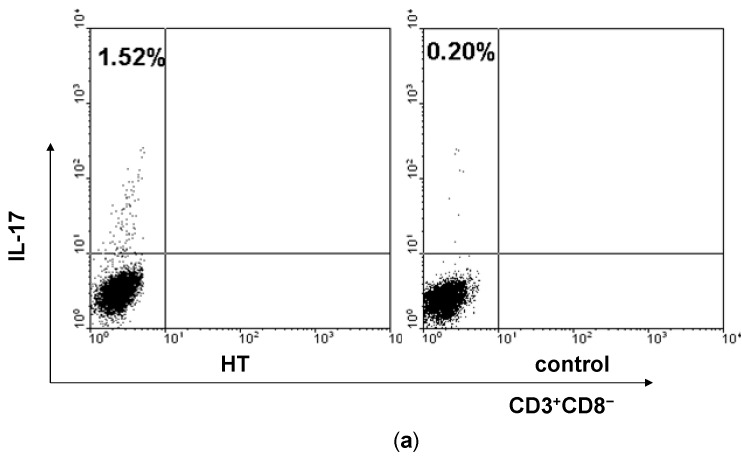

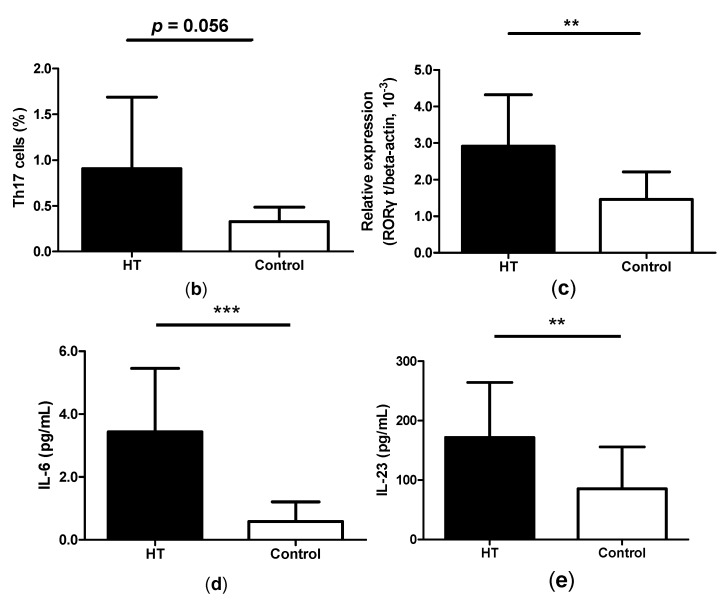
Enhancement of Th17 cells in peripheral blood from HT patients. PBMCs from HT patients and healthy controls were incubated with PMA/ionomycin, stained for cell surface ijms CD3 and CD8 as well as intracellular IL-17 and analyzed by flow cytometry. (**a**) Representative dot plots from HT patient and healthy control are shown. Values correspond to the percentage of Th17 cells. We used isotype controls to determine the positive cells, and all the values are gated on the CD3^+^CD8^−^ cells; (**b**) Percentages of Th17 cells were compared between HT patients and healthy controls; (**c**) Levels of ROR-γt mRNA in PBMCs from HT patients and healthy controls; (**d**) Serum levels of IL-6 were determined by ELISA in serum samples from HT patients and healthy controls and (**e**) Serum levels of IL-23 were determined by ELISA in serum samples from HT patients and healthy controls. ** *p* < 0.01; *** *p* < 0.001.

Firstly, we analyzed the percentage of Th17 cells in PBMCs of HT patients by flow cytometry. Because the stimulation of PMA/ ionomycin could down-regulate the expression of human CD4 molecule, we selected CD3^+^CD8^−^ as a marker for CD4 T cells according to several previous studies [25,26]. We gated on CD3^+^CD8^−^ in PBMCs and identified IL-17^+^ cells to distinguish the Th17 cells from T cells in PBMCs (Figure 1a). It was found that HT patients showed an increase of Th17 cells at the border of statistical significance (*p* = 0.056, Figure 1b).

We next measured mRNA expression levels of ROR-γt in PBMCs, which plays a considerable role in differentiation of Th17 cells [27]. Compared with the healthy control, HT patients had significantly higher ROR-γt mRNA levels (Figure 1c).

It was reported that IL-6 and IL-23 are essential in the differentiation of Th17 cells [28,29,30]. In order to clarify the influencing factors of Th17 cells enhancement in HT patients, we analyzed the levels of IL-6 and IL-23 in serum from HT patients and healthy controls. We found that HT patients have significantly increased serum concentration of IL-6 and IL-23 in comparison with healthy controls (Figure 1d,e).

### 2.2. Reduction of Regulatory T Cells in Peripheral Blood from HT Patients

**Figure 2:**
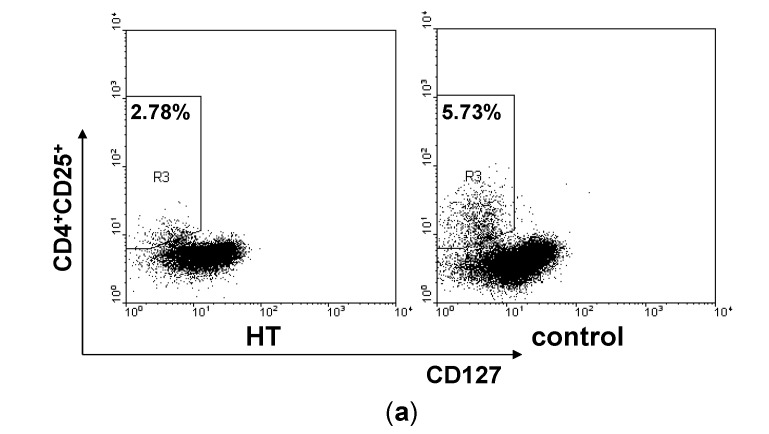

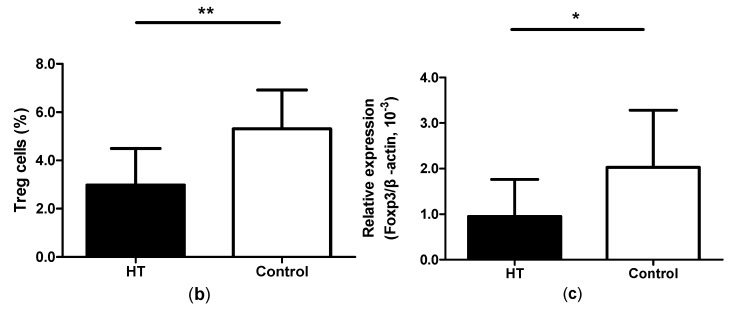
Reduction of regulatory T cells in HT patients. PBMCs from HT patients and healthy controls were stained for cell surface molecule CD4, CD25, CD127 and analyzed by flow cytometry. (**a**) Representative dot plots from HT patients and healthy controls are shown. Values correspond to the percentage of Treg cells. We used isotype controls to determine the positive cells, and all the values are gated on the CD4^+^ cells; (**b**) Percentages of Treg cells were compared between HT patients and healthy controls and (**c**) Levels of Foxp3 mRNA in PBMCs from HT patients and healthy controls. * *p* < 0.05; ** *p* < 0.01.

Subsequently, we gated on CD4^+^CD25^+^CD127^low^ T cells in PBMCs to distinguish Treg cells in the peripheral blood (Figure 2a). The proportion of Treg cells was reduced in PBMCs from patients with HT compared with healthy controls (Figure 2b). Foxp3 is the transcription factor of Treg cells [31]. qRT-PCR analysis displays an attenuated expression of Foxp3 mRNA in the PBMCs from HT patients (Figure 2c).

### 2.3. Correlation between Th17/Treg Balance and TgAb Levels in HT Patients

**Figure 3:**
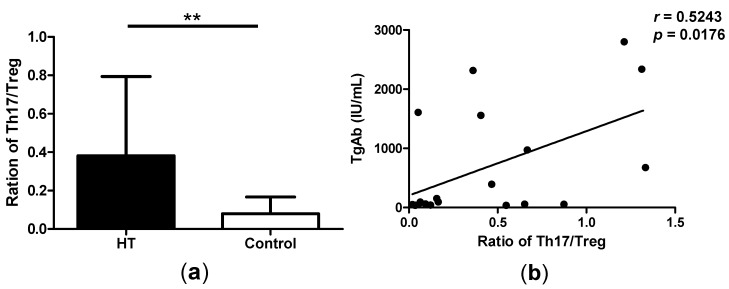
Correlation between Th17/Treg balance and TgAb levels in HT patients. (**a**) The ratio of Th17/Treg in patients with HT compared with healthy controls and (**b**) The correlation between the ratio of Th17/Treg and TgAb levels in HT patients. ** *p* < 0.01.

It has reported that, the control of Th17/Treg balance appears very important in the development of autoimmune diseases. We found that the ratio of Th17/Treg is significant higher in patients with HT compared with healthy controls (Figure 3a). In patients with HT, the levels of autoantibodies such as TgAb are upregulated, and it is one of the most important indexes of prognosis for HT. So we analyzed the correlation of TgAb levels and the ratio of Th17/Treg. By flow cytometry analysis, we found that the Th17/Treg ratio correlated positively with the TgAb levels (Figure 3b). This result demonstrated that the ratio of Th17/Treg could reflect the severity of HT in some extent.

### 2.4. High Levels of GITRL in Patients with HT

**Figure 4:**
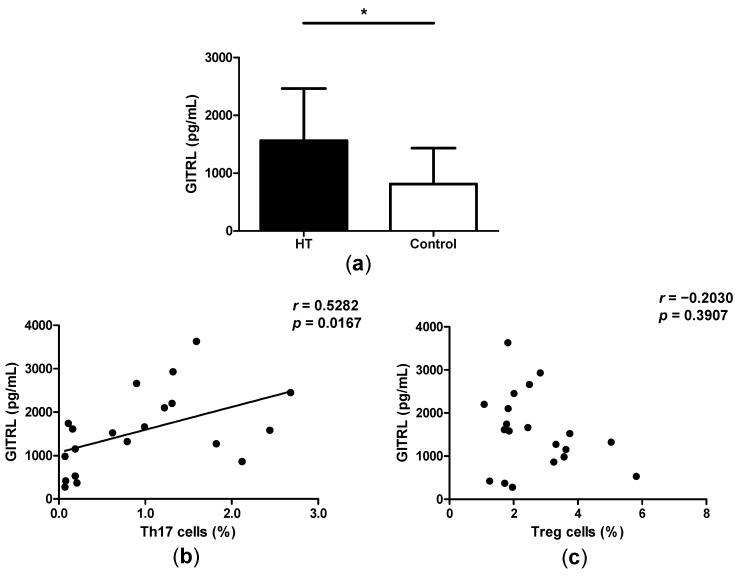
High levels of GITRL in patients with HT. (**a**) Serum levels of GITRL were determined by ELISA in serum samples from HT patients and controls; (**b**) The correlation between the percentage of Th17 cellsand serum levels of GITRL in HT patients and (**c**) The correlation between the percentage of Treg cells and serum levels of GITRL in HT patients. * *p* < 0.05.

Previous study has revealed that Foxp3^+^CD4^+^CD25^+^T regulatory cell (nTreg)-mediated suppression of immune response is abrogated following combination of glucocorticoid-induced tumor necrosis receptor (GITR) family-related protein. Additionally, we have confirmed that GITRL could enhance the levels of Th17 cells in mice, and aggravate collagen-induced arthritis in mice [11]. In this study we have demonstrated the increased ratio of Th17/Treg in HT patients, and a positive correlation between the ratio of Th17/Treg and TgAb levels. We detected the levels of GITRL in the serum of HT patients by ELISA and found that GITRL levels are significantly augmented in the serum of HT patients as compared to healthy controls (Figure 4a). Also, there was a positive correlation between the levels of GITRL and the proportion of Th17 cells (Figure 4b). Apart from this, there is no correlation between the levels of GITRL and the percentages of Treg cells in HT patients (Figure 4c).

### 2.5. Increased Expression of IL-17, ROR-γt and GITRL mRNA in Thyroid Tissue from HT Patients

**Figure 5:**
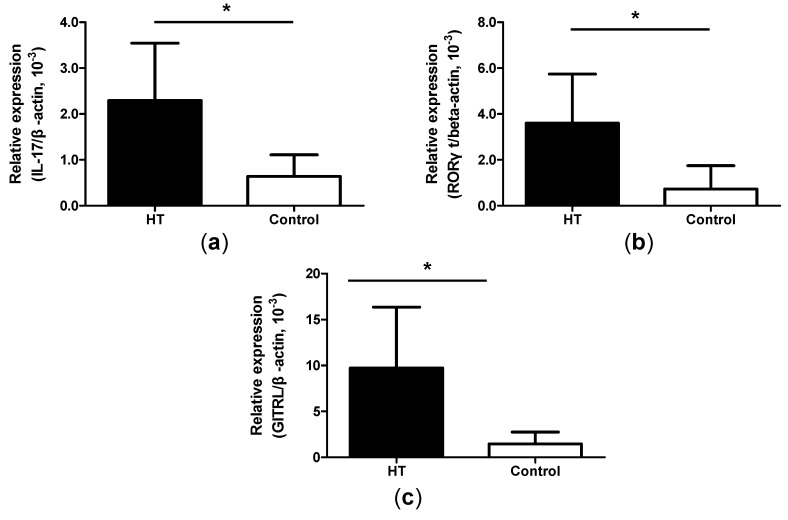
Higher expression of IL-17, ROR-γt and GITRL mRNA in thyroid tissue from HT patients. (**a**) The levels of IL-17 mRNA in thyroid glands were detected by real-time PCR from HT patients, and patients with simple goiter; (**b**) The levels of ROR-γt mRNA in thyroid glands were detected by real-time PCR from HT patients, and patients with simple goiter and (**c**) The levels of GITRL mRNA in thyroid glands were detected by real-time PCR from HT patients, and patients with simple goiter. * *p* < 0.05.

HT is an organ-specific immune disease, and lymphoid infiltration in thyroid tissue is a significant feature. In order to investigate the thyroid tissue, we collected six thyroid glands of HT patients. Real-time PCR showed increased expression of IL-17 and ROR-γt mRNA in thyroid glands from HT patients compared with simple goiter patients (Figure 5a,b). Apart from these, the expression of GITRL mRNA in the thyroid glands from HT patients was significantly higher when compared with simple goiter patients (Figure 5c).

## 3. Discussion

The pathological basis of HT so far remains largely unexplored. Some studies have shown that HT is a Th1-mediated autoimmune disease. Besides Th1 cells, some other subsets of CD4^+^T cells participate in the process of several autoimmune diseases, such as Th17 cells and Treg cells. These two kinds of CD4^+^T cells have opposite roles in the development of autoimmune diseases. While Th17 cells promote autoimmunity, Treg cells can control it. This study seeks to provide insights into the involvement of Th17/Treg cells in the pathogenesis of HT.

Previous studies have found the enhancement of Th17 cells and its related cytokines in HT patients [32,33,34,35]. Apart from this, the critical function of Tregs in the process of HT have been elucidated in two recent manuscripts [36,37]. However, these studies did not clarify the balance between Th17 and Treg cells, in addition to the possible factors involved in this phenomenon. Based on this speculation, we assessed the proportion of Th17 and Treg cells in the peripheral blood of 25 patients with HT and 20 healthy controls. We identified CD3^+^CD8^−^IL-17^+^ cells to distinguish the Th17 cells from PBMCs. Increased Th17 cells were found in PBMCs from patients with HT. The expression of IL-17 mRNA was upregulated in the thyroid glands of HT patients. And there was an increased expression of ROR-γt mRNA in PBMCs as well as in the thyroid glands of HT patients. The levels of IL-6 and IL-23, which play an important role in the differentiation of Th17, were increased in HT patients’ serum. Apart from this, we identified CD4^+^CD25^+^CD127^low^ cells to distinguish Treg cells from PBMCs. As expected, the percentage of CD4^+^CD25^+^CD127^low^ cells was decreased in PBMCs of HT patients. The expression of Foxp3 mRNA in PBMCs from HT patients was also attenuated. These data confirmed the important role of Th17 and Treg cells in the pathogenesis of HT. In addition to this, the ratio of Th17/Treg was significantly higher in HT patients, indicating that the imbalance of Th17/Treg may be involved in the development of HT.

It is widely accepted that the upregulation of autoantibodies such as TgAb is the most objective clinical manifestations of HT; it could indicate the development of HT and prognosis for this disease. We found an increasing ratio of Th17/Treg in HT patients in the present study. We thus analyzed the correlation of TgAb levels and the high ratio of Th17/Treg. A positive correlation was found between TgAb levels and the ratio of Th17/Treg. These results collectively indicate that the imbalance of Th17/Treg could reflect the severity of HT to some extent.

GITR is a type 1 transmembrane protein induced on T cells following TCR stimulation or by the glucocorticoid dexamethasone [16,38]. It is expressed at different levels in CD4^+^ and CD8^+^T cells, CD4^+^CD25^+^Tregs constitutively express high levels of GITR on the cell surface [17,18]. The natural ligand of GITR, GITRL, is predominantly expressed by antigen-presenting cells [19]. It has been reported that the engagement of GITR by GITRL abrogated the immunosuppressive function of Treg cells [18]. GITRL could also reinforce autoimmune arthritis via the enhancement of the expansion of Th17 cells [8]. To further analyze the imbalance of Th17/Treg in HT patients, we detected the levels of GITRL in the serum of HT patients and the expression of GITRL mRNA in the thyroid glands from HT patients. The levels of GITRL were significantly increased in the serum of HT patients as compared to healthy controls, and the expression of GITRL mRNA in the thyroid glands from HT patients was significantly higher when compared with patients with simple goiter either. Also, there was a positive correlation between the levels of GITRL and the proportion of Th17 cells. But there is no correlation between the levels of GITRL and the percentages of Treg cells in HT patients. According to recent studies, the CD4^+^CD25^low^GITR^+^ cell is a novel human CD4^+^ T-cell population with regulatory activity and expanded in patients with Sjögren’s syndrome or SLE [39,40,41]; the results of these studies provide new insight for our follow-up study.

In summary, our data suggest that increases in Th17 cells may be caused by increased levels of GITRL, and that the high ratio of Th17/Treg could participate in the pathopoiesis of Hashimoto’s thyroiditis.

## 4. Experimental Section

### 4.1. Individuals and Samples

Twenty-five patients with Hashimoto’s thyroiditis were included in this study. The diagnosis was based on commonly accepted classification criteria. All the patients were on the period of onset of HT at the time of this study. Main clinical data of these patients are shown in Table 1. Peripheral blood samples were obtained from all patients. The serum concentrations of anti-TgAb and TPO-Ab were detected by chemiluminescent immunoassay (MAGLUMI 2000 PLUS, Shenzhen New Industries Biomedical Engineering Co., Shenzhen, China). All samples were taken in accordance with the regulations and approval of the Affiliated People’s Hospital of Jiangsu University.

**Table 1:** Clinical features of HT patients and healthy controls enrolled in the study.

	HT Patients	Healthy Controls	Range
Number	25	20	
Gender (M/F)	5/20	3/17	
Age (year)	47.2 ± 9.9	46.3 ± 7.2	
Tg-Ab (IU/mL)	677.1 ± 312.3	19.4 ± 9.3	<30
TPO-Ab (IU/mL)	317.5 ± 206.7	16.6 ± 7.1	<10

Data correspond to the arithmetic mean ± SD.

### 4.2. Cell Isolation and Stimulation in Vitro

Peripheral blood mononuclear cells (PBMCs) were isolated by density-gradient centrifugation over Ficoll-Hypaque solution. After isolation PBMCs were incubated in complete RPMI 1640 culture medium in the presence of 1.0 μg/mL ionomycin and 50 ng/mL phorbol myristate acetate (PMA; Sigma-Aldrich, St. Louis, MO, USA) for 5 h. After 5 h of culture at 37 °C under 5% CO_2_, cells were collected and centrifuged for Th17 cells and mRNA detection by flow cytometric analysis and qRT-PCR, respectively. Unstimulated PBMCs were used for the staining of Treg cells.

Thyroid specimens were minced and then digested with collagenase II (Sigma-Aldrich, St. Louis, MO, USA) for 1–2 h at 37 °C, then these cells were isolated by density-gradient centrifugation over Ficoll-Hypaque solution. Thyroid mononuclear cells (TMC) were obtained and cell viability was more than 95%.

### 4.3. Flow Cytometric Analysis

Unstimulated PBMCs were washed and immunostained with fluorescein isothiocyanate-conjugated anti-CD4 (eBioscience, San Diego, CA, USA), phycoerythrin (PE)-conjugated anti-CD25 (eBioscience, San Diego, CA, USA), phycoerythrin-cy5-conjugated anti-CD127 (eBioscience, San Diego, CA, USA) mAb against human cells surface. Stimulated PBMCs were immunostained with phycoerythrin-cy5-conjugated anti-CD3 (eBioscience, San Diego, CA, USA), fluorescein isothiocyanate-conjugated anti-CD8 (eBioscience, San Diego, CA, USA), phycoerythrin (PE)-conjugated anti-IL-17 (eBioscience, San Diego, CA, USA) mAb against human cells. All the staining was according to manufacturers’ protocol. Isotype-matched Ab controls were used in all procedures. Cells were analyzed using a FACSCalibur flow cytometer and CELLQUEST software (Becton Dickinson, Sparks, MD, USA). The results were expressed as the percentage of CD4^+^CD25^+^CD127^low^ cells and CD3^+^CD8^−^ cells expressing IL-17.

### 4.4. RNA Isolation and Real-Time PCR

For the detection of cytokine IL-17, transcription factor RORC and Foxp3, PBMCs were stimulated for 5 h as described above. After that activated cells were used to quantify the expression of IL-17, RORγt and Foxp3mRNA by real-time PCR. TRIzol reagent (Invitrogen, Carlsbad, CA, USA) was added in stimulated PBMCs. After isolated total RNA, reverse transcription was performed according to the manufacturer’s instruction (Toyobo, Osaka, Japan). Real-time PCR was performed in duplicate using Bio-Rad SYBRGreen super mix (Bio-Rad, Hercules, CA, USA). Primer sequences were as follows: IL-17, sense, 5'-CAAGACTGAACACCGACTAAG-3'; antisense, 5'-TCTCCAAAGGAAGCCTGA-3', RORγt, sense, 5'-CCTGGGCTCCTCGCCTGACC-3'; antisense, 5'-TCTCTCTGCCCTCAGCCTTGCC-3', Foxp3, sense, 5'-ACAGCACATTCCCAGAGTTCCT-3'; antisense, 5'-Rev:TCTCCACCCGCACAAAGCA-3'.

We used β-actin as internal control. Data were analyzed by Bio-Rad CFX Manager software.

### 4.5. Cytokine Quantification

Levels of IL-6, IL-23 (eBioscience, San Diego, CA, USA) and GITRL (R&D Systems, Inc., Minneapolis, MN, USA) were determined by ELISA method using an ELISA reader (μQuant, BIO-TEK, Winooski, VT, USA). All determinations were performed by duplicate and the lower detection limits for IL-6, IL-23 and GITRL were 1.5625, 15.625 and 125 pg/mL respectively.

### 4.6. Statistical Analysis

Student’s unpaired *t*-test was performed to determine whether there was a statistically significant change between two groups. Correlations between variables were determined by Spearman’s correlation coefficient. Data were analyzed with GraphPad Prism5 software (GraphPad Software, Inc., San Diego, CA, USA).

## Abbreviations

GITRL: glucocorticoid-induced TNF receptor family-related protein ligand
HT: Hashimoto’s thyroiditis.
